# Case-Fatality Rates and Sequelae Resulting from *Neisseria meningitidis* Serogroup C Epidemic, Niger, 2015

**DOI:** 10.3201/eid2210.160731

**Published:** 2016-10

**Authors:** Matthew E. Coldiron, Halidou Salou, Fati Sidikou, Kadadé Goumbi, Ali Djibo, Pauline Lechevalier, Idrissa Compaoré, Rebecca F. Grais

**Affiliations:** Epicentre, Paris, France (M.E. Coldiron, R.F. Grais);; Epicentre, Niamey, Niger (H. Salou);; Centre de Recherche Médicale et Sanitaire (CERMES), Niamey (F. Sidikou);; Ministry of Public Health, Niamey (K. Goumbi, A. Djibo);; Médecins Sans Frontières, Paris (P. Lechevalier);; Médecins Sans Frontières, Niamey (I. Compaoré)

**Keywords:** *Neisseria meningitidis*, meningitis, meningococcal, Serogroup C meningococcal meningitis, bacteria, gram-negative bacteria, epidemics, complications, Niger

## Abstract

We describe clinical symptoms, case-fatality rates, and prevalence of sequelae during an outbreak of *Neisseria meningitidis* serogroup C infection in a rural district of Niger. During home visits, we established that household contacts of reported case-patients were at higher risk for developing meningitis than the general population.

A novel strain of *Neisseria meningitidis* serogroup C has been circulating in parts of the African meningitis belt since 2013 (*1*), causing a large-scale epidemic in Nigeria and Niger in 2015. This novel strain appeared after the introduction of a conjugate vaccine (PsA-TT, MenAfriVac) against *N. meningitidis* serogroup A, previously the major cause of epidemic meningitis in the region. *N. meningitidis* serogroup A has been virtually eliminated as a result of PsA-TT, although the first cases of *N. meningitidis* serogroup C in Nigeria in 2013 predated MenAfriVac introduction there. We describe case-fatality rates and sequelae resulting from infection with *N. meningitidis* serogroup C in a rural health district of Niger.

## The Study

During January 1–June 30, 2015, a total of 562 suspected cases of meningitis and 51 deaths (case-fatality ratio [CFR] 9.1%) from this disease were reported in the national surveillance system in the Dogondoutchi health district of Niger (estimated population 727,282) (*2*). Médecins Sans Frontières supported Niger’s Ministry of Public Health in its epidemic response in Dogondoutchi by providing patient care with 5 days of injectable ceftriaxone, as described in recent recommendations (*3*); reinforcing surveillance by providing training on case definitions; collecting data; and collaborating in reactive vaccination campaigns. A patient-level database containing patient demographics, treatment, laboratory results, and outcome was maintained during January 2–May 17, 2015; during this period, the database compiled information for 473 case-patients. In September 2015, in collaboration with the Ministry of Health, teams consisting of a community health worker and a nurse attempted home visits to each of the 473 case-patients (or surviving family members) in the database. Team members verified patients’ vital status and treatments received and assessed for presence of 6 major sequelae, offering treatment and referral when necessary. Paralysis and anosmia were noted on physical exam (anosmia was evaluated by asking participants to smell a pungent local food with their eyes closed). Persistent convulsions, hearing loss, loss of developmental milestones (among children <5 years of age), and persistent mental incapacity (among persons >5 years of age) were subjectively reported by patients (or family members). During the home visit, teams used a standardized questionnaire to collect information and conduct a household census (Table). Team members asked whether meningitis had been diagnosed in other family members during the epidemic and verified reported diagnoses by written records available from the patient or by verbal confirmation of the number of doses of intramuscular ceftriaxone received. A meningitis death was considered to be any death occurring <30 days of symptom onset.

**Table:** Characteristics of 369 suspected meningitis patients visited at home after the epidemic season, Dogondoutchi, Niger, September 2015

Characteristic	No. (%) patients
Sex	
M	220 (59.6)
F	149 (40.4)
Age, y	
<2	22 (6.0)
2–4	57 (15.5)
5–14	190 (51.5)
15–29	84 (22.8)
30–44	13 (3.5)
>45	3 (0.8)
Positive by PCR	194 (62.2)
* Neisseria meningitidis* serogroup C	144 (74.2)
* N. meningitidis* serogroup W	36 (18.6)
* Streptococcus pneumoniae*	12 (6.2)
* N. meningitidis* serogroup unspecified	2 (1.0)
Delay between symptom onset and visit to health center, d*	
0	90 (24.4)
1	176 (47.7)
2	63 (17.1)
3	23 (6.2)
≥4	14 (0.8)

*Data missing for 3 patients.

The original patient database was anonymized during analysis, and no identifying information was collected. Niger’s Ministry of Public Health approved the overall intervention, including the home visits conducted after the epidemic. All medical care was free of charge to patients.

Of the 473 meningitis patients in the database, 369 (78.0%) could be visited at home. In the original patient database, 54 deaths were recorded (CFR 11.4%). During the home visits, 6 patients that were reported as dead in the database were found to be alive; 22 patients reported as recovered had in fact died, for a total of 70 meningitis deaths (CFR 14.8%).

Collection of biologic samples for confirmation was emphasized during the epidemic; consequently, 406 (82.5%) of the 473 reported patients received a lumbar puncture, and 252 (63.6%) patient samples were positive for *N. meningitidis*, *Streptococcus pneumoniae*, or *Haemophilus influenzae* type b infection by PCR performed at the national reference laboratory for meningitis in Niamey. Among the positive samples, 189 (75.0%) were *N. meningitidis* serotype C; 47 (18.7%) were *N. meningitidis* serotype W; 13 (5.2%) were *S. pneumoniae*; and 3 (1.2%) were *N. meningitidis* serogroup unspecified. 

Of the 189 patients with confirmed *N. meningitidis* serogroup C infection, 23 died (CFR 12.2%). A difference in death rates by sex was not statistically significant: 12 (9.8%) of 123 male patients died, compared with 11 (16.7%) of 66 female patients (p = 0.16 by χ^2^ test). No difference appeared between recorded clinical signs and symptoms (i.e., headache, fever, neck stiffness, convulsions, vomiting, irritability, altered consciousness, bulging fontanelles, and focal neurologic symptoms) for patients with confirmed *N. meningitidis* serotype C infection and those for patients with all other suspected infections.

During the follow-up home visits, surviving patients were evaluated for sequelae. Among patients with suspected cases, prevalence of any sequela was 10.8%. The rate of sequelae among patients with confirmed *N. meningitidis* serogroup C infection was 15.1% (19/126), compared with 7.7% (3/39) for patients with all other confirmed infections and 7.9% (11/140) for all other patients (i.e., with suspected but unconfirmed cases or with suspected cases with negative test results) (p = 0.06). The 2 most common reported sequelae among patients with confirmed infections were hearing loss (15/126 [11.9%]) and persistent mental incapacity or loss of milestones (4/126 [3.2%]). Patients with these sequelae did not differ in sex or age from other patients. 

The 369 case-patients (or their surviving family members) who received home visits after the epidemic lived in 346 households. For 298 households, only 1 case occurred; 48 (13.0%) households had multiple case-patients. Among these 48 households, all case-patients in 22 households appeared in the patient database. However, the home interviews revealed 26 households with additional case-patients (i.e., who confirmed receiving multiple doses of ceftriaxone) not found in the patient database. Taking into account the household census, the attack rate for subsequent case-patients in a household after notification of a first case was 1,760/100,000 population. During the same period, in the Dogondoutchi health district, the overall attack rate was 79/100,000 population. Median time between onset of the first case in a household and a subsequent case was 3 days (interquartile range 1–8).

## Conclusions

By collecting detailed information on individual notified cases and then making home visits to ≈80% of case-patients 4 months after the epidemic ended, we gathered insights into this novel strain of *N. meningitidis* serogroup C. The CFR in the Dogondoutchi district was higher than the country’s average and was also higher among women. Although slightly higher than CFRs seen in past *N. meningitidis* serogroup A epidemics, deaths recorded in the surveillance system were mostly in line with those occurring in historic epidemics. The prevalence of sequelae in confirmed cases of *N. meningitidis* serogroup C also falls in line with descriptions of sequelae caused by other meningococcus serotypes (*4*). Overall, the characteristics of this epidemic are similar to those of historic epidemics of *N. meningitidis* before the introduction of MenAfriVac (*4*–*6*).

Home visits enabled quantification of attack rates among members of households reporting a meningitis case. Because of the short time between the first and subsequent cases, subsequent cases should not be considered secondary cases, although household members are at higher risk for infection compared with the community at large. This finding could eventually support the use of antimicrobial drug prophylaxis for household members of meningitis patients, although more research on this topic is warranted. Available data did not permit subdistrict-level analyses. Given the heterogeneous distribution of cases across districts in other epidemics (*7*), subdistrict-level analyses would be preferable to district-level analyses.

Finally, even with dedicated resources to reinforce surveillance during the epidemic, we found unreported cases during the home visits and, after confirmation of patients’ vital status, the case-fatality ratio differed from that reported. Our findings indicate that even reinforced surveillance systems are not perfectly sensitive, but incomplete reporting should not deter continued case-based surveillance.
